# Variation within *MBP* gene predicts disease course in multiple sclerosis

**DOI:** 10.1002/brb3.670

**Published:** 2017-03-09

**Authors:** Yuan Zhou, Steve Simpson, Jac C. Charlesworth, Ingrid van der Mei, Robyn M. Lucas, Anne‐Louise Ponsonby, AUSLONG Investigators Group, Bruce V. Taylor

**Affiliations:** ^1^ Menzies Institute for Medical ResearchUniversity of TasmaniaHobartTASAustralia; ^2^ National Centre for Epidemiology and Population HealthCanberraACTAustralia; ^3^ Murdoch Childrens Research InstituteUniversity of MelbourneMelbourneVICAustralia

**Keywords:** clinically definite MS, expanded disability status scale, genetics and single‐nucleotide polymorphism, myelin basic protein, relapse

## Abstract

**Objective:**

Prognosis following a first demyelinating event is difficult to predict, with no genetic markers of MS progression currently identified. Myelin basic protein (MBP) is a major component of the myelin sheath of CNS neurons and may play a central role in demyelinating diseases such as MS. However, genetic variation in *MBP* has not been implicated in MS onset risk in large genome‐wide association studies. We hypothesized that genetic variations in *MBP* may be a determinant of MS clinical course.

**Materials and Methods:**

We investigated whether variations in the *MBP* gene altered clinical course (conversion to MS and/or relapse, and annualized change in disability), using a prospectively collected longitudinal cohort study of 127 persons who had had a first demyelinating event, followed up to the 5‐year review.

**Results:**

We found one variant, rs12959006, predicted worse clinical outcomes. The risk genotype (CT + TT) was significantly associated with hazard of relapse (HR = 1.74, 95% CI = 1.19–2.56, *p *= .005) and of greater annualized disability progression (β = 0.18, 95% CI = 0.06–0.30, *p *= .004). We also found a significant interaction between the risk genotype and baseline anti‐HHV6 IgG in predicting MS (*p*_interaction_ = 0.05) and relapse (*p*_interaction_ = 0.02). Functional prediction analysis showed this variant is the target of many transcription factors and the binding sites of miR‐218 and miR‐188‐3p.

**Conclusions:**

Our results provide novel insights into the role of genetic variation within the *MBP* gene predicting MS clinical course, both directly and by interaction with known environmental MS risk factors.

## 1 Introduction

Multiple sclerosis is a complex inflammatory and neurodegenerative disease of the central nervous system (CNS) whose causation and progression are determined by both genetic and environmental factors (Compston & Coles, 2008). CNS inflammation is associated with active degradation of the myelin sheath (Compston & Coles, 2008). Myelin basic protein (MBP) is a major component of the myelin sheath and is believed to play an important role in the process of myelination in the CNS (Deber & Reynolds, 1991). In patients presenting with a first demyelinating event (FDE), the presence of serum antibodies against MBP significantly predicted conversion to multiple sclerosis (MS) in some (Berger et al., 2003; Tomassini et al., 2007) but not all studies (Berger & Reindl, 2007; Kuhle et al., 2007).

Emerging evidence suggests that autoimmune responses targeting the myelin sheath indicates the existence of myelin‐reactive T cells, which can recognize MBP peptides, trigger an immune response against MBP and thus affect the integrity of the myelin sheath (Stinissen & Hellings, 2008). The molecular mimicry hypothesis suggests that these myelin‐reactive T cells can be activated as certain viral agents, particularly human herpesvirus‐6 (HHV6) and Epstein–Barr virus (EBV), share similar antigenic profiles with MBP, resulting in T cells cross‐reacting to both virus and MBP (Holmoy & Vartdal, 2005; Tejada‐Simon, Zang, Hong, Rivera, & Zhang, 2003).

Despite its potential role in MS, none of the large genome‐wide association studies (GWAS)(International Multiple Sclerosis Genetics C, 2013; International Multiple Sclerosis Genetics C, Wellcome Trust Case Control C, 2011) have found any variants in MBP that predict MS risk. By their nature GWAS studies are not well‐suited to predicting clinical course, as they use a case–control design to define risk. In fact, no MS GWAS has been able to clearly establish any markers that predict severity or clinical course. Therefore, candidate gene approaches with *a priori* hypotheses can be used to avoid the burden of correcting for the large number of tests associated with GWAS studies.

Given the high prevalence of prior exposure and seropositivity (>90%) against HHV6 (Soldan et al., 1997) and EBV (Pender & Burrows, 2014) in the general population, and the existence of myelin‐reactive T cells in healthy individuals (Pette et al., 1990), we have therefore hypothesized that by using a biologically plausible candidate gene approach, that genetic variations in MBP may directly, or by interaction with HHV6 or EBV, determine clinical outcomes (conversion to MS after a first demyelinating event, relapse rate, and disability). We have therefore studied this *a priori* hypothesis in a well‐characterized cohort referred soon after a well‐described FDE, and who had both genetic data and anti‐EBV and anti‐HHV6 antibody serology.

## 2 Material and Methods

### 2.1 Study design

The Ausimmune Longitudinal (AusLong) Study, which built upon the original Ausimmune case–control study, seeks to elucidate environmental, genetic, and personal risk factors for the onset and early progression of MS. This study has followed 169 cases with a classical first demyelination event (FDE)(Lucas et al., 2007). The present analysis is for the period from first recorded symptom onset, to the 5‐year review, as this is the most recent face‐to‐face review which all currently enrolled participants have completed.

The AusLong Study was approved by nine regional Human Research Ethics Committees. All participants gave written informed consent.

### 2.2 Exposure and clinical course measures

Several clinical outcomes were evaluated, including conversion to MS, occurrence of relapse and annualized disability progression from FDE to 5‐year review.

Conversion to MS was defined primarily as the occurrence of two or more clinical demyelinating episodes, thus satisfying the diagnostic requirements of dissemination in space and time, or a single episode plus paraclinical evidence, as per the 2005 McDonald criteria (Polman et al., 2011) (a minority of cases were diagnosed following MRI based on this latter criterion [*n *= 20]). Conversion to MS was reported at annual review and cross‐checked with neurological records and review of MRI scans. Study‐specific standardized MRI scans were undertaken at the 2/3‐year time point for those who had not converted clinically and for all cases at 5 years and were used to define McDonald 2005 definite MS.

A relapse was defined according to the 2001 McDonald Criteria (McDonald et al., 2001) as the acute or subacute appearance or reappearance of a neurological abnormality (lasting at least 24 hr) in the absence of other potential explanatory factors. Relapses were reported at annual review and only relapses which were diagnosed and verified by a neurologist were included in the analysis. Disability was assessed by the Kurtzke Expanded Disability Status Scale (EDSS) (Kurtzke, 1983), assessed at the 5‐year review; the EDSS on the day before FDE was assumed to be 0 as no case reported preexisting neurological dysfunction.

Clinical history was recorded by the study neurologist with additional information derived from medical records at initial presentation, describing the nature of the episode/symptoms which brought the participant into the Ausimmune Study, as well as historical symptoms prior to presentation. The presence of prior neurological symptoms thought by the assessing neurologist to constitute at least probable demyelination excluded these cases from this analysis as did a progressive course at presentation. A full treatment history with disease‐modifying drugs was also recorded annually.

Key EBV antigens (Anti‐EBNA‐1 IgG, anti‐EBNA‐2 IgG) and HHV 6 antigen (anti‐HHV6 IgG) titers were measured in serum samples collected at baseline, using immunofluorescence assays: Anti‐EBNA1 commercial ELISA (DiaSorin), anti‐EBNA2 in‐house ELISA, and anti‐HHV6 commercial ELISA (PanBio) as previously described (Strautins et al., 2014).

### 2.3 Genotyping

DNA from AusLong participants was genotyped using the Illumina customized MS exome genotyping array (Illumina Human Exome‐12 v1.2 array (~244,000 SNPs) plus additional MS relevant variants (~87,000) added as a customized component). Genotypes were called using Illumina GenomeStudio software. Individuals were excluded for the following reasons: a call rate of <99% or duplicate discordance. Variants were excluded on the basis of a call rate of <99% or a deviation from Hardy–Weinberg equilibrium with *p *< 1.0 × 10^−6^. Principal components analysis was carried out twice, once excluding HapMap samples to identify population outliers and once including HapMap samples to help interpret outliers (Guo et al., 2014). All eight genotyped tagSNPs (rs9676113, rs3794832, rs7232502, rs12959006, rs61742988, rs3900176, rs11150997, and rs7233242) with *r* ^2^ < .1 in the MBP gene (chr18: 74690789–74844774) were included in the analysis.

### 2.4 Data analysis

Predictors of time to conversion to MS and of relapse were evaluated by Cox proportional hazards regression models, the latter for repeated events using the gap‐time model by Prentice and colleagues (Prentice, Williams, & Peterson, 1981). All covariates satisfied the proportional hazards assumption, excepting study site in the relapse analysis. Accordingly all relapse analyses were stratified on study site.

Predictors of annualized change in EDSS were evaluated using linear regression, adjusted for whether persons were having a relapse at the time of their 5‐year EDSS assessment. Because the annualized change in EDSS was highly skewed, a log‐transformation was applied to satisfy linear regression assumptions of minimal heteroskedasticity. All means and coefficients, however, were back‐transformed and presented on the original scale of the change in EDSS variable.

Interaction was assessed by generating a product term of the two variables to be assessed, with the *p*‐value of this two‐component term delineating the significance of the interaction.

All statistical analyses were conducted in Stata/SE 12.1 (StataCorp LP, College Station, TX, USA).

## 3 Results

Of the 169 case participants in the Ausimmune/AusLong Study that had a classic FDE, 127 have undergone genotyping as described and had been assessed at 5 years and form the cohort assessed in this study. They were predominantly female (*n *= 98, 77.2%), age at study entry (mean: 37.8, SD: 9.5), conversion to MS (*n *= 68, 53.5%), relapse number (*n *= 152), and 5‐year EDSS (median: 1, IQR: 0–2).

### 3.1 Risk genotype (CT + TT) of rs12959006 directly predicts progression to relapse and annualized change in EDSS

**Figure 1:**
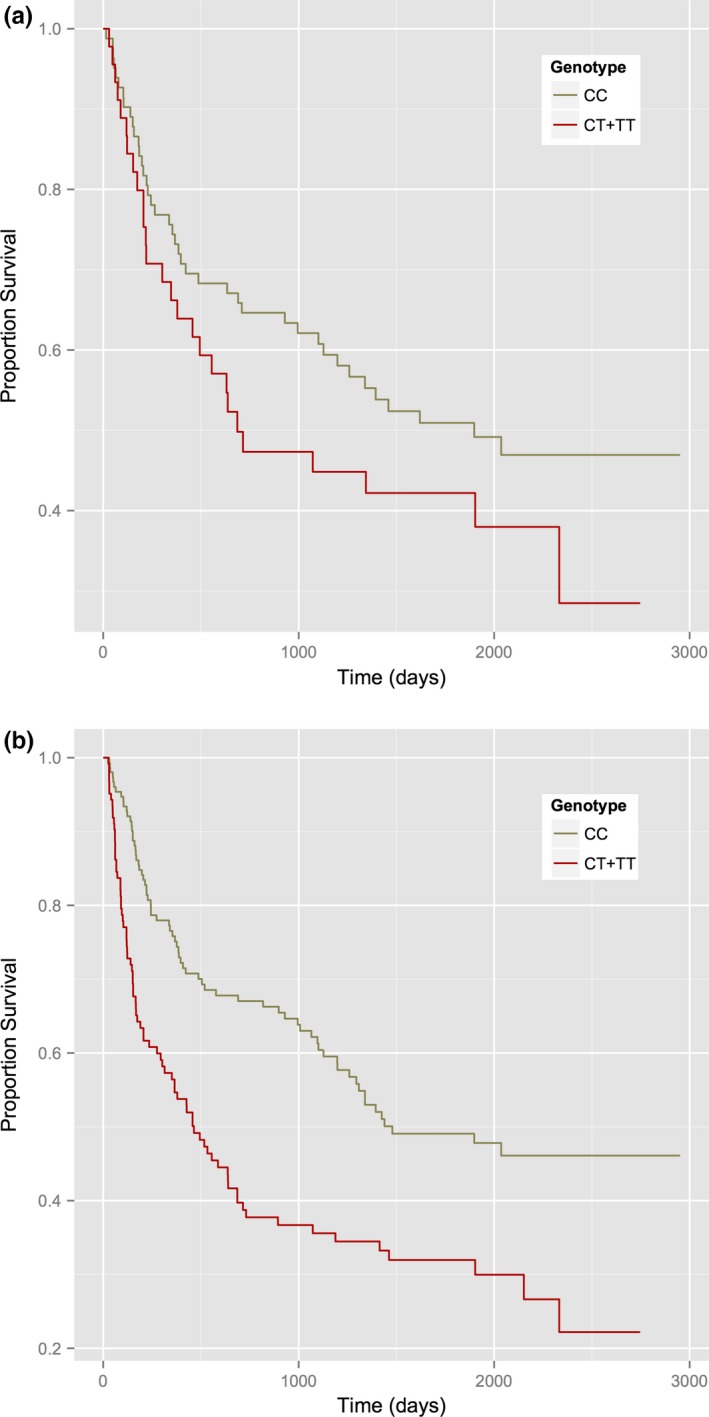
(a) Kaplan–Meier survival plot for time to CDMS by category of rs12959006 genotype. (b) Kaplan–Meier survival plot for time to relapse by category of rs12959006 genotype

Among the group of participants with a classic FDE and a second event diagnostic of MS within the period after referral to the study, only two persons had the TT genotype of rs12959006. Therefore, the homozygous risk genotype was combined with the heterozygote as CT + TT. We found the genotype (CT + TT) of rs12959006 showed a trend to association with risk of conversion to MS compared to the CC genotype (HR = 1.57 (95% CI: 0.93–2.64), *p *= .09). Examining this SNP in predicting relapse, a stronger and statistically significant result was seen (HR = 1.74 (95% CI: 1.19–2.56), *p *= .005, Table 1), partly reflecting the greater number of events (*n *= 68 MS vs. 152 relapses). Figures 1a and b show the survival curves for the rs12959006 genotype for time to conversion to MS and relapse, respectively.

**Table 1:** rs12959006 predicting CDMS, relapse and annualized ΔEDSS in MS among participants with a classic FDE

rs12959006	*N*	CDMS	*N*	Relapse	*N*	ΔEDSS
CC	41	Ref	70	Ref	37	0.26 (0.20, 0.32)
CT + TT	27	1.57 (0.93, 2.64)	82	**1.74 (1.19, 2.56)**	24	**+0.18 (0.06, 0.30)**
		*p *= .09		***p *** **= .005**		***p *** **= .004**

HR, hazard ratio; CI, confidence interval; β, beta coefficient; EDSS, Expanded Disability Status Scale; FDE, first demyelinating event.

Due to the small number people carrying TT genotype who converted to CDMS (*N *= 2), we recoded the genotype as CT + TT. Genotype CC was used as reference in the analysis. Results were adjusted for age, sex and study site, and presented as HR (95% CI) for time to CDMS and relapse. Disability results are presented as geometric mean annualized disability progression (95% CI) for the reference group, whereas coefficient relative to reference (β [95% CI]) are presented for subsequent levels. N refers to the number of events for each related clinical course. p value <0.05 bolded.

Evaluating the association of rs12959006 with annualized change in EDSS, the CT + TT genotype was associated with a significantly greater rate of disability progression, with 0.18 greater annualized EDSS increase per year (β = 0.18, 95% CI: 0.06–0.30, *p *= .004). Translating this to a clinical outcome, those carrying the CT + TT genotype will have an EDSS score 0.9 points greater over 5 years than those carrying the CC genotype.

We did not observe any association between the other seven MBP SNPs and progression to MS, relapse or annualized change in EDSS (data not shown). After adjusting for multiple testing ((*p *= .05/8)≈0.006 was defined as significant), rs12959006 remained significant in predicting relapse and annualized ΔEDSS. The effect of the risk allele rs12959006 was in the same direction when predicting all three clinical outcomes and was toward a more active clinical course, providing further support for a true effect.

A minority of cases had received some form of disease‐modifying therapies (DMT) between FDE and conversion to MS (*n *= 24) including treatment as a covariant in the models did not alter the outcomes. Almost all cases received some form of treatment post conversion to MS and again this did not alter associations.

### 3.2 rs12959006 genotype interacts significantly with baseline anti‐HHV6 IgG levels to predict conversion to MS and relapse

As shown in Table 2, we found a significant interaction between the rs12959006 genotype and baseline anti‐HHV6 IgG levels. In those with the risk genotype (CT + TT), there was a significant positive association between anti‐HHV6 IgG and time to MS (HR = 6.95, 95% CI: 1.11–43.31, *p *= .04; *p*_interaction_ = 0.05) and relapse (HR = 3.00, 95% CI: 1.19–7.53, *p *= .02; *p*_interaction_ = 0.02), whereas there was no association among the nonrisk allele carriers (CC) (HR = 0.63, 95% CI: 0.14–2.77, *p *= .54, for time to MS and HR = 0.58, 95% CI: 0.20–1.64, *p *= .30 for time to relapse). There was no significant interaction between rs12959006 genotype and baseline anti‐ENBA‐1 or anti‐EBNA‐2 IgG titers for time to MS or relapse, although the effect was in the same direction as for HHV6. rs12959006 genotype did not significantly interact with baseline anti‐HHV6, anti‐EBNA1, or anti‐EBNA2 IgG levels in predicting annualized ΔEDSS.

**Table 2:** rs12959006 interaction with baseline‐measured anti‐HHV‐6, anti‐EBNA‐1, and anti‐EBNA‐2 IgG to predict CDMS and relapse among classic FDEs

Factor	Genotype	CDMS		*p*	Relapse		*p*
		*N*	HR (95% CI)		*N*	HR (95% CI)	
Baseline HHV6	CC	23	0.63 (0.14, 2.77)	.54	27	0.58 (0.20, 1.64)	.30
	CT + TT	16	**6.95 (1.11, 43.31)**	**.04**	**23**	**3.00 (1.19**,** 7.53)**	**.02**
			***p***_**interaction**_	**.05**		***p***_**interaction**_	**.02**
Baseline EBNA1	CC	23	1.30 (0.57, 2.99)	.54	27	1.10 (0.57, 2.12)	.77
	CT + TT	16	**3.01 (1.15, 7.87)**	**.02**	23	1.28 (0.88, 1.87)	.19
			*p*_interaction_	.28		*p*_interaction_	.66
Baseline EBNA2	CC	23	1.12 (0.59, 2.14)	.73	27	1.13 (0.74, 1.73)	.57
	CT + TT	16	**4.14 (1.62, 10.56)**	**.003**	23	1.52 (0.90, 2.56)	.12
			*p*_interaction_	.19		*p*_interaction_	.68

HR, hazard ratio; CI, confidence interval; FDE, first demyelinating event.

Due to the smaller number people carrying TT genotype converted to CDMS (*N *= 2), we recoded the genotype as CT + TT. Results were adjusted for age, sex and study site, and presented as HR (95% CI) for CDMS and relapse. N refers to the number of events for each related clinical course. p value <0.05 bolded.

Functional prediction analysis (Xu & Taylor, 2009) showed this variant rs12959006 is the target of many transcription factors and the binding sites of miR‐218 and miR‐188‐3p.

## 4 Discussion

We have shown that people with an FDE who carry the risk SNP of rs12969006 in *MBP* have a worse outcome on key measures of disease progression (relapse rate and EDSS progression), and that the risk SNP of rs129569006 within the *MBP* gene interacts with serological markers of prior HHV6 infection to predict clinical course post FDE. Past infection with HHV6 and EBV are both well‐recognized risk factors for MS onset and there is some evidence that HHV6 IgG levels in particular are associated with MS progression (Simpson et al., 2012).

MBP undergoes complex posttranscriptional modification, including methylation, phosphorylation, and miRNA binding (Harauz et al., 2004). The risk locus studied is a target for many transcription factors as well as the binding sites of miR‐218 and miR‐188‐3p based on functional prediction (Xu & Taylor, 2009). Other research has shown that miR‐218 expression is significantly down regulated in MS white matter compared to controls (Noorbakhsh et al., 2011). However, the exact molecular mechanisms by which changes in miR‐218 and this MBP variant may modify myelination and demyelination remains unknown, with further studies need.

Importantly we have shown that in the post‐GWAS MS world, where the focus has shifted from defining risk associations to defining determinants of clinical course, studying *a priori* hypotheses such as those we have described, can be undertaken successfully in moderate sized, well‐characterized longitudinal cohorts, using data on multiple aspects of MS clinical course and potential genetic and environmental factors.

There are several potential caveats to our findings. By their nature, longitudinal cohort studies are at best of moderate sample size, and further subdivision into those with particular phenotypes and exposure parameters decreases the power to detect associations. Additionally, within this cohort we do not have data on change in MRI metrics such as lesion load and brain volume which have been shown to predict MS outcomes post FDE.

On the other hand, the internal consistency across the three outcome measures, the biologically plausible directions of effect and interactions with herpesvirus, as well as the dose‐dependency of effect in our disability analysis, is evidence supporting a true association rather than statistical artifact (Tabor, Risch, & Myers, 2002). Still, validation of our findings in other longitudinal cohorts is essential.

These data demonstrate that genetic variants in MS candidate genes with biologic plausibility may help predict MS clinical course. Additionally, interaction with well‐documented serological markers of prior EBV and HHV6 infection enhance these effects and further supports the notion of complex gene‐environment interactions in the onset and progression of MS. These results, if replicated, may aid in developing prognostic algorithms in the early disease period in MS, as well as providing further mechanistic insights.

## Conflicts of Interest

The authors declare that they have no potential conflicts of interests.
